# Prearrest vital sign abnormalities are associated with adverse outcomes in pediatric ICU cardiac arrest: a get with the guidelines-resuscitation analysis

**DOI:** 10.1016/j.resuscitation.2025.110846

**Published:** 2025-09-24

**Authors:** Sanjiv D. Mehta, Cody-Aaron Gathers, Lindsay N. Shepard, Mary Putt, Nadir Yehya, Kathryn Graham, Ryan W. Morgan, Robert M. Sutton

**Affiliations:** a Department of Anesthesiology and Critical Care Medicine? Children’s Hospital of Philadelphia, United States; b Department of Biostatistics, Epidemiology and Informatics, Perelman School of Medicine at the University of Pennsylvania, United States

**Keywords:** Pediatrics, Cardiac arrest, Vital signs, Physiology, Pediatric intensive care unit, Cardiac intensive care unit

## Abstract

**Aim:**

We aimed to quantitatively describe vital sign abnormalities prior to pediatric IHCA and evaluate whether the severity of abnormalities was independently associated with survival.

**Methods:**

In a retrospective cohort study using the American Heart Association’s Get with The Guidelines-Resuscitation^®^ registry, children with ≥1 min of cardiopulmonary resuscitation (CPR) in an Intensive Care Unit (ICU) from 2007 to 2022 with prearrest vital signs were included. Vital signs most proximate to CPR (10–120 min prior) were classified as abnormal (HR or RR >95th, SBP or DBP <5th percentile for age). Multivariable regression adjusted for age, illness category, prearrest conditions, and prearrest interventions assessed the associations between vital sign abnormalities and outcomes (primary: survival to hospital discharge, secondary: return of spontaneous circulation [ROSC]).

**Results:**

Of 2875 IHCA patients meeting inclusion criteria, 1790 (62.3 %) had at least one abnormal vital sign. Patients with vital sign abnormalities were older, had non-surgical illness categories, and higher prevalence of prearrest illnesses and interventions. Low SBP (<5%) was the vital sign with the lowest odds of survival to hospital discharge (aOR 0.56 [95 %CI 0.46–0.68], p < 0.01) and ROSC (aOR 0.63 [95 %CI 0.54–0.73], p < 0.01). There was a stepwise decrease in the adjusted odds of survival for each additional abnormal vital sign (1 vs 0: aOR 0.62 [95 %CI 0.51–0.76], p < 0.01; 2 vs 1: 0.72 [95 %CI 0.53–0.97] p = 0.03; 3 vs 2: 0.53 [95 %CI 0.33–0.86] p < 0.01).

**Conclusions:**

Prearrest vital sign abnormalities are common in pediatric ICU IHCA and independently associated with worse outcomes, emphasizing the need for prompt detection and intervention to improve outcomes.

## Introduction

Pediatric in-hospital cardiac arrest (IHCA) affects more than 15,000 children annually, with survival rates of 40–50 %.^1–3^ Most pediatric IHCA occur in intensive care units (ICUs), where continuous car diorespiratory monitoring enables real-time response to rapidly changing physiology.^1,4^ Physiology during cardiopulmonary resuscitation (CPR) and post-arrest care is well characterized, and specific findings (e.g., hypotension) are associated with worse survival rates.^1,5,6^ Single center studies have explored prearrest physiology.^7,8^ However, the relationship between specific vital sign abnormalities and resuscitation outcomes has not been systematically quantified in a large, multicenter cohort of critically ill children. Understanding the relevance of prearrest vital sign abnormalities may help clinicians effectively recognize intervenable *peri*-arrest physiology.

To investigate the relationship between prearrest physiology and resuscitation outcomes in critically ill children, we leveraged the American Heart Association Get With The Guidelines-Resuscitation^®^ (GWTG-R) registry to: (1) characterize the prevalence and patterns of prearrest vital sign abnormalities in pediatric ICU IHCA, and (2) evaluate whether specific vital sign derangements and combinations of abnormalities are associated with survival to hospital discharge and return of spontaneous circulation (ROSC). We hypothesized that vital sign abnormalities would be associated with decreased rates of survival and ROSC, even after accounting for illness severity indicators and prearrest interventions.

## Methods

### Study design

This study was a retrospective cohort study of prospectively collected data from the GWTG-R quality improvement registry. It was deemed exempt by the Children’s Hospital of Philadelphia Institutional Review Board (IRB 23–021731).

GWTG-R is a national quality improvement registry that uses an Utstein-style template for uniform reporting of cardiac arrest events at participating institutions.^9,10^ Over 100 sites report on IHCAs in neonates and children. Details on sites, data collection procedures, and reliability have been previously described.^9,11–13^ The optional prearrest vital sign data field allows sites to enter up to four sets of vital signs from the 4 h prior to arrest (Supplementary Fig. 1).

### Subjects

Hospitalized children (0–18 years) receiving ≥1 min of CPR in a pediatric or cardiac ICU between 2007 and 2022 were included. Only the first or “index” IHCA for each patient’s hospitalization was included. Analysis was restricted to ICUs because >90 % of pediatric IHCAs occur there^1^ and consistent physiologic monitoring provides the most feasible setting for prearrest information to inform decision-making. Cardiac arrests in neonatal ICUs were not included due to unique characteristics of this population and distinct CPR practices.^14^

Cases missing time of arrest, outcomes, or pre-specified confounders including illness category (e.g., medical or surgical, cardiac or non-cardiac) and prearrest conditions were excluded. Cases lacking at least one complete set of valid vital signs (heart rate (HR), respiratory rate (RR), systolic blood pressure (SBP), and diastolic blood pressure (DBP) at a single time point) in the 10 to 120 min prior to arrest were also excluded. The 10 to 120 min before arrest time-frame avoided immediate peri-arrest deterioration or onset of cardiac arrest while including physiology that was clinically relevant to the arrest. Vital signs were valid if they were physiologically plausible (HR <300 and >1 beats per minute, RR <150 and >1 breaths per minute, SBP <300 and >1 mmHg, DBP <200 and >1 mmHg, and DBP < SBP). Finally, cases from sites at which less than 50 % of eligible IHCA cases per year had documented vital signs were excluded. This exclusion minimized bias from inconsistent reporting, while maintaining site representation.

### Variables

#### Vital sign data

If patients had multiple valid vital sign sets, only the most proximate set was used. Reported vital signs were converted to age-based percentile categories for valid comparison across ages.^15,16^ Vital signs were categorized as abnormal (HR/RR >95th, SBP/DBP <5th) and severely abnormal (HR/RR >99th, SBP/DBP <1st) based on pre-specified thresholds. Our analysis focused on common physiologic precursors of arrest (tachycardia, tachypnea, hypotension), while excluding conditions that are themselves an indication for CPR (e.g. bradycardia with poor perfusion). Supplementary Tables 1 and 2 provide the age-based percentile thresholds for abnormal and severely abnormal vital signs.

#### Outcomes

The primary outcome was survival to hospital discharge. Sustained ROSC (>20 min) was a secondary outcome. Survival with good neurologic outcome was not evaluated due to inconsistent reporting (32 % of survivors missing data).

### Statistical methods

The statistical analysis plan, including primary, exploratory, and sensitivity analyses were specified prior to examination of the data. Patients with and without any abnormal signs were compared using descriptive statistics. Categorical variables are reported as counts with frequencies and compared using Chi-Square tests. Continuous variables are reported as medians with interquartile ranges and compared using Wilcoxon rank-sum tests. Additionally, included patients were compared to those meeting inclusion criteria but excluded for missing vital sign data. Site characteristics were compared for sites meeting versus not meeting vital sign reporting criteria.

To evaluate the association of abnormal vital signs and outcomes, multivariable regression with generalized estimating equations (GEE) was used with an independent correlation structure and robust covariance matrix to account for dependencies among patients within hospitals. We chose a GEE model to estimate the population-averaged effect, which aligns with our research question of understanding broad trends in a national registry. This approach is also robust to the varied hospital cluster sizes present in our data. The primary analysis considered the association of the presence of an individual abnormal vital sign (binary yes/no) with outcomes. Because multiple abnormalities may represent increasingly abnormal physiology, the cumulative impact of multiple abnormal vital signs on outcomes was also evaluated. The number of abnormal vital signs was modeled as a categorical variable allowing for non-linear associations between the exposure and outcome. Because systolic and diastolic blood pressures are highly correlated, an abnormality in either measure was counted as a single abnormal vital sign. Thus, a patient could have a maximum of three abnormal vital signs at one time.

Models were adjusted for covariates with established or hypothesized associations with both prearrest vital signs and survival: age^17^ (continuous variable), hospital illness category^2,18^ (medical cardiac, surgical cardiac, medical non-cardiac, or surgical-cardiac), presence of prearrest conditions^19–21^ (all yes/no: hypotension/hypoperfusion, respiratory insufficiency, sepsis, pneumonia, and metabolic or electrolyte abnormalities), and presence of prearrest medical interventions (all yes/no: vasoactive infusions, invasive ventilation). Age was included in the model alongside age-based vital sign percentile categories to account for any residual confounding on outcomes as it has been previously shown to be associated with survival after pediatric cardiac arrest.^17^ Finally, we also included prearrest conditions not specified above with >10 % prevalence in the cohort.

Exploratory analyses examined: 1) outcome associations with specific combinations of abnormal vital signs (HR + RR, HR + SBP or DBP, RR + SBP or DBP, and narrow pulse pressure: SBP-DBP < 20 mmHg) as exposures; 2) outcome associations with only severely abnormal vital signs (HR or RR >99th and SBP or DBP <1st) as exposures; and 3) outcome associations with each vital sign percentile category (e.g., HR in the 50–95 %, 95–99 %, >99) as exposures. Predicted probabilities of outcomes for each percentile category were estimated using a GEE model with vital sign percentile as the exposure and adjustment for the same covariates as the primary analysis. Pairwise differences in outcomes between categories were compared using Wald tests. A Bonferroni adjustment (0.05/k comparisons) controlled the family-wise error rate for pairwise comparisons. The category with the highest predicted probability of survival or ROSC served as the reference group.

For binary exposures, results are presented as odds ratios with 95 % confidence intervals (CI). For categorical exposures, results are presented as predicted probabilities with CI. Statistical analyses completed in Python (version 3.11.0, Python Software Foundation, Wilmington, Delaware) using the Statsmodel package.^22,23^

### Sensitivity analyses

Sensitivity analyses evaluated result robustness using alternative vital sign selection criteria. To capture more remote vital sign abnormalities, associations with the earliest recorded vital signs (10 min – 4 h prearrest) were assessed. To explore bias from excluding incomplete vital sign sets or vital signs from low reporting sites, the association of any abnormal recorded vital signs (10 min 4 h prearrest) were assessed. Here, any single recorded vital sign meeting abnormal criteria was included (e.g., a patient with a single HR reported in the 95 % would be classified as having an abnormal HR).

## Results

### Description of the cohort

**Fig. 1 –:**
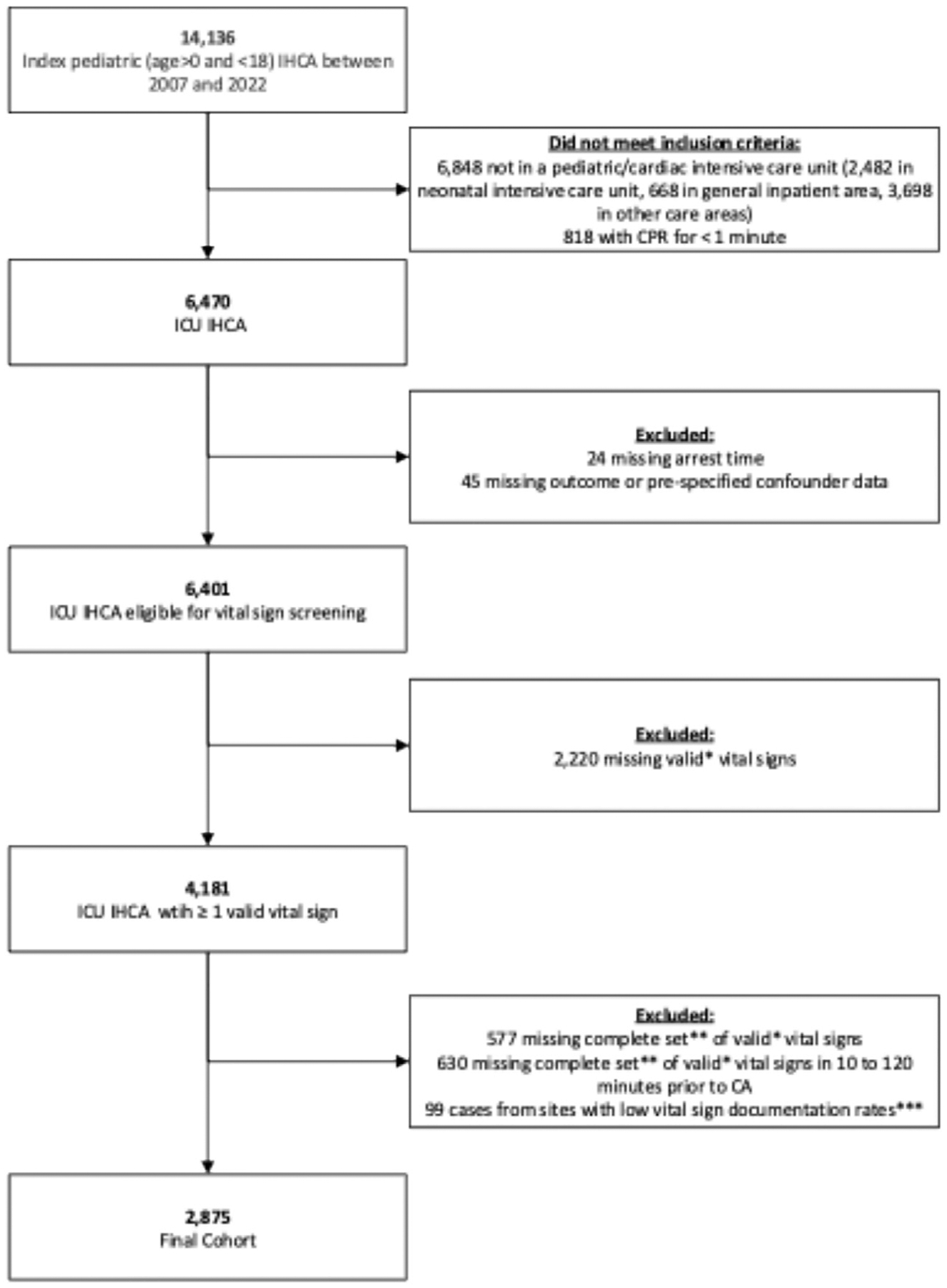
Study Cohort Flowchart: Application of Inclusion and Exclusion Criteria. Application of inclusion and exclusion criteria to arrive at final study cohort of 2875 events. * valid defined as vital signs in physiologic range: heart rate >1 and <300 beats per minute, respiratory rate >1 and <150 breaths per minute, systolic blood pressure >1 and <300 mmHg, diastolic blood pressure >1 and <200 mmHg. ** complete set defined as having all four vital sign values (heart rate, respiratory rate, systolic blood pressure, and diastolic blood pressure) recorded at the same time. *** from sites with low vital sign documentation rates defined as sites at which less than 50 % of eligible In-hospital cardiac arrest (IHCA) cases per year had documented vital signs.

Of 6470 patients with index in-ICU IHCA events, 2875 (44.4 %) met study criteria with at least one set of valid prearrest vital signs (Fig. 1). Of these, 1790 (62.3 %) had at least one abnormal vital sign. Children with abnormal vital signs, versus none, were older (median 2.0 [IQR 0.6, 10.0 vs 0.4 [IQR 0.2, 2.0], p < 0.01) and more often had medical non-cardiac illness category (52 % vs 44.9 %, p < 0.01) (Table 1). They more frequently had prearrest hypotension (35.3 % vs 17.6 %, p < 0.01), metastatic disease (9.2 % vs 3.0 %, p < 0.01), and sepsis (19.1 % vs 9.2 %, p < 0.01). They more often received vasoactive agents prior to arrest (60.6 % vs 47.2 %, p < 0.01). They were more often pulseless at the onset of CPR (51.7 % vs 40.0 %, p < 0.01) and received longer median durations of CPR (median 14.0 [IQR 5.0, 36.0] vs 10.0 [3.0, 29.0], p < 0.01). Additional prearrest conditions with > 10 % prevalence in the cohort prompting inclusion in multivariable modeling were: cyanotic cardiac malformations (22.5 % vs 27.8 %, p < 0.01), acyanotic cardiac malformations (14.2 % vs 18.8 %, p < 0.01), non-cardiac congenital malformations (15.6 % vs 20.6 %, p < 0.01), baseline depressed central nervous system (16.3 % vs 16.1 %, p = 0.97), and renal insufficiency (13.9 % vs 8.6 %, p < 0.01).

Patients excluded due to missing outcome data or vital sign data differed significantly from included patients across several demographic and prearrest conditions (Supplementary Table 3). Most differences were small (standardized mean differences [SMD] of <0.1) though statistically significant due to sample size. Of variables with >0.1 SMD, excluded patients had fewer cyanotic cardiac malformations or vasoactive agents at the time of arrest, and more often had “other” illness category. Excluded patients were more often pulseless at CPR start, had lower rates of ROSC, and had lower rates of survival, although all of these had SMD <0.1.

Of 145 eligible sites, 85 (59.3 %) consistently reported vital signs and were included. Included sites more frequently had >100 pediatric beds, contributed more arrests, reported more arrests per year, and more frequently documented vital signs for events (94.7 % vs 4.7 %, p < 0.01) (Supplementary Table 4).

The median time from the recorded vital sign included in the primary analysis to start of CPR was 40 [IQR 21, 65] minutes. The distribution of when the most proximate vital signs were recorded, stratified by their abnormality status, is shown in Supplementary Fig. 2. Patients had a median of 2 [IQR 1, 2] sets of vital signs documented in the prearrest period. Table 2 describes the frequency and composition of vital sign abnormalities. Of the 1790 patients with any abnormal vital sign, 1148 (64 %) had at least one severely abnormal vital sign (Table 2). Abnormal blood pressure (SBP or DBP) was the most common abnormal vital sign (1160/1790, 64.8 %) and severely abnormal vital sign (691/1148, 60.2 %). Abnormal HR and RR were more prevalent in cases with more than one abnormal vital sign – more often occurring with another abnormal vital sign rather than having extreme values in isolation.

### Association of abnormal vital signs and outcomes

Patients with at least one abnormal vital sign less frequently achieved ROSC (1061/1790 [59.3 %] vs. 835/1085 [77.0 %], p < 0.01), more frequently received extra-corporeal CPR (ECPR) (258/1790 [14.4 %] vs. 113/1085 [10.5 %], p < 0.01), and less frequently survived to hospital discharge (689/1790 [38.5 %] vs. 667/1085 [61.5 %], p < 0.01).

#### Primary outcome: Association between abnormal vital signs and survival to hospital discharge

**Fig. 2 –:**
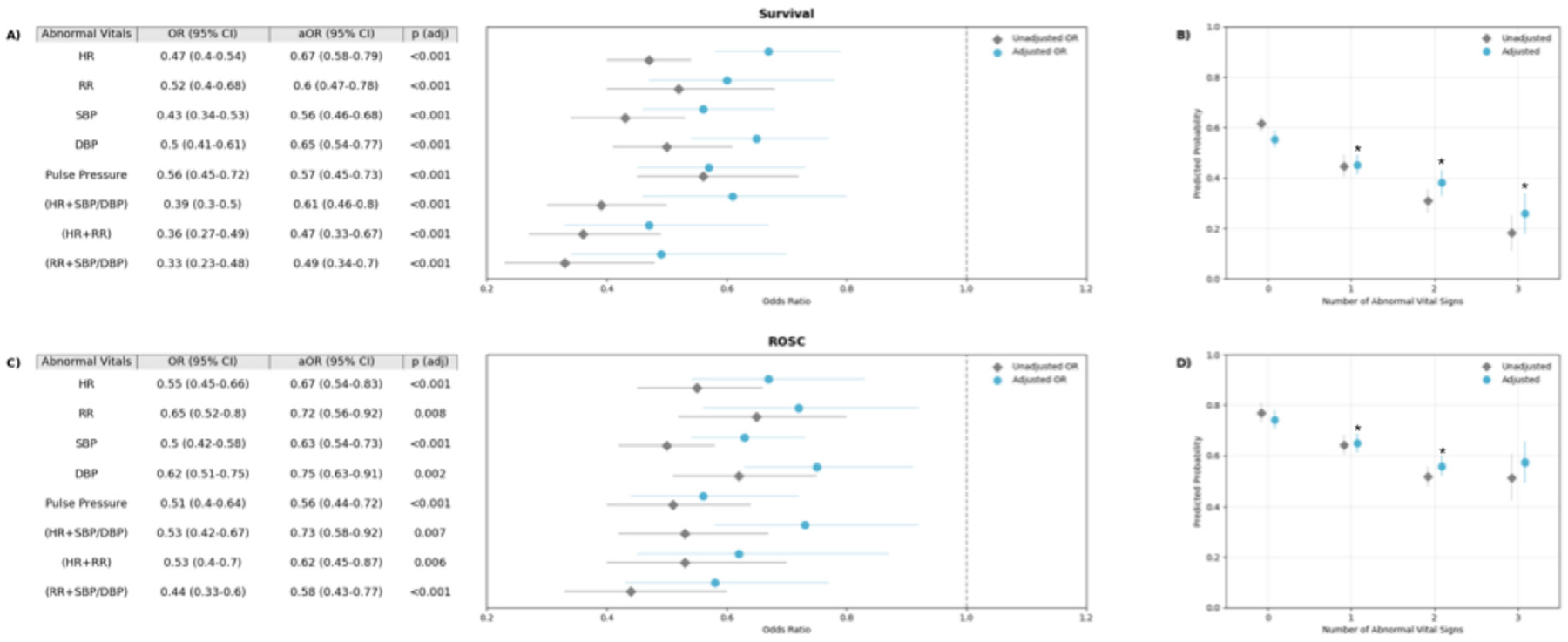
Association between presence of abnormal vital signs with outcomes. Forrest plots (A and C) show the association between abnormal vital sign exposures for survival (A) and ROSC (C). Adjusted odds ratios (aOR) account for age, illness category, prearrest conditions, and ICU intervention. Error bars represent the 95 % confidence interval. P-values report the results of the adjusted odds ratios. Panels B and D show the association between the number of abnormal vital signs and predicted probability of survival (B) and ROSC (D) unadjusted and after adjustment for age, illness category, prearrest conditions, and ICU intervention. Error bars represent the 95 % confidence interval. * Indicates a significant difference between that number of abnormal vital signs and the prior group (e.g., 1 vs 0 abnormal vital signs, 2 vs 1, or 3 vs 2) for the adjusted model. HR = Heart rate (abnormal defined as >95 % for age). RR = Respiratory rate (abnormal defined as >95 % for age). SBP = Systolic blood pressure (abnormal defined as <5 % for age), DBP = Diastolic blood pressure (abnormal defined as <5 % for age). Pulse pressure = Difference between SBP and DBP (abnormal defined as <20 mmHg).

The associations between specific vital signs abnormalities and survival to hospital discharge are presented in Fig. 2. The presence of each type of vital sign abnormality was associated with lower odds of survival to hospital discharge after controlling for confounders. Amongst individual vital signs, the lowest odds ratio was observed for abnormal SBP (aOR 0.56 [95 %CI 0.46–0.68], p < 0.01) (Fig. 2, Panel A). There was a stepwise decrease in the adjusted odds of survival for each additional abnormal vital sign (1 vs 0 abnormal vital signs: aOR 0.62 [95 %CI 0.51–0.76], p < 0.01; 2 vs 1: 0.72 [95 %CI 0.53–0.97] p = 0.03; 3 vs 2: 0.53 [95 %CI 0.33–0.86] p < 0.01) (Fig. 2, panel B).

#### Secondary outcome: Association of abnormal vital signs and ROSC

Each individual vital sign abnormality was similarly associated with lower odds of ROSC, and abnormal SBP had the lowest odds of ROSC (aOR 0.63 [95 %CI 0.54–0.73], p < 0.01) (Fig. 2, Panel C). There was a stepwise decrease in the adjusted odds of ROSC for each additional abnormal vital sign up to 2 abnormal vital signs (1 vs 0 abnormal vital signs: aOR 0.63 [95 %CI 0.49–0.81], p < 0.01; 2 vs 1: aOR 0.67 [95 %CI 0.52–0.88], p < 0.01; 3 vs 2: aOR 1.1 [95 %CI 0.72–1.6], p = 0.75) (Fig. 2, Panel D). Supplementary Table 5 details the adjusted odds ratios for all covariates in the final multivariable models.

### Exploratory analyses

Of specified combinations of vital sign abnormalities, abnormal HR and RR together had the lowest odds of survival (aOR 0.47 [95 % CI 0.33–0.67], p < 0.01) and narrow pulse pressure had the lowest odds of ROSC (aOR 0.56 [95 %CI 0.44–0.72], p < 0.01) (Fig. 2, Panels A and C).

The relationship between severely abnormal vital signs and outcomes followed a similar pattern to the primary analysis (Supplementary Fig. 3). Severely abnormal SBP had the lowest odds of survival (aOR 0.51 [95 %CI 0.40–0.65], p < 0.01) and ROSC (aOR 0.63 [95 %CI 052–0.76], p < 0.01). Severely abnormal vital signs had a similar stepwise decrease in survival with each additional vital sign (1 vs 0 abnormal vital signs: aOR 0.54 [95 %CI 0.45–0.66], p < 0.01; 2 vs 1: aOR 0.73 [95 %CI 0.55–0.96], p = 0.02; 3 vs 2: aOR 0.79 [95 %CI 0.23–2.74], p = 0.71).

**Fig. 3 –:**
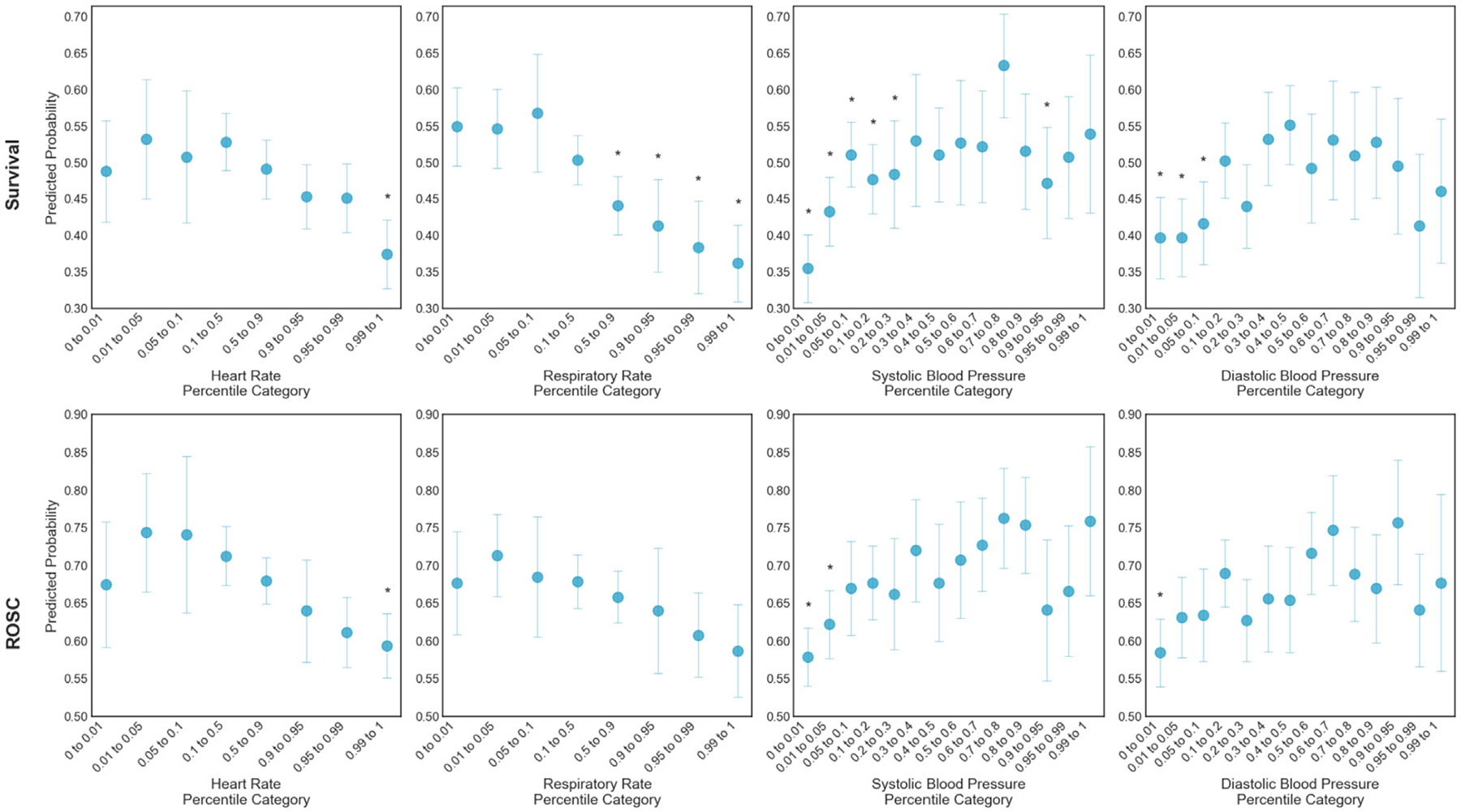
Association between vital signs grouped by percentiles of age-based normalized values and outcomes. Predicted probability of survival (top row) and ROSC (bottom row) across age-based percentile categories for each vital sign. Predicted probabilities estimated from a multivariable logistic regression adjusted for age, illness category, prearrest conditions, and ICU interventions. Survival = survival to hospital discharge. ROSC = sustained return of spontaneous circulation (>20 min after arrest). * Denotes a significant difference from the reference group (highest probability of outcome) using a Bonferroni corrected significance threshold to account for multiple comparisons (0.05/13).

Fig. 3 illustrates the adjusted association between individual vital sign percentile categories and outcomes from the multivariable model. Higher HR and RR percentile categories had lower probabilities of survival, with the lowest predicted probability of survival for the highest percentile groups (>99 %) (HR: 37.4 % [95 %CI 32.7–42.2 %]; RR: 36.2 % [95 %CI 30.9–41.4 %]). Lower SBP and DBP percentile groups had lower probabilities of survival, with the lowest predicted probabilities of survival at the lowest percentile groups (<1%) (SBP: 35.5 % [95 %CI 30.8–40.1 %]; DBP: 39.7 % [95 %CI 34.1–45.2 %]). Variability in ROSC for each vital sign was less pronounced but followed a similar relationship. The lowest predicted probability of survival was also at the most extreme percentile groups (>99 % for HR and RR, <1% for SBP and DBP) (HR: 59.4 % [95 %CI 55.2–63.5 %]; RR: 58.7 % [95 %CI 52.6–64.8 %]; SBP: 57.9 % [95 % CI 54.1–61.7 %]; DBP:58.5 % [95 %CI 53.9–63.0 %]).

### Sensitivity analyses

In sensitivity analyses including the earliest recorded vital signs in the 10 min to 4 h prearrest and non-simultaneously recorded vital signs, associations between abnormal vital signs and outcomes were similar to those identified in the primary analysis (Supplementary Figs. 4 and 5).

## Discussion

In this multicenter observational study of pediatric ICU IHCA using the AHA GWTG-R registry, prearrest vital sign abnormalities were associated with adverse outcomes. More than 60 % of children with cardiac arrest had at least one abnormal vital sign before arrest and each additional abnormal vital sign was associated with progressively lower odds of survival to hospital discharge. These associations persisted after adjusting for prearrest illnesses, ICU interventions, illness category, and age. This multicenter study linking prearrest physiologic status to CPR outcomes underscores the critical need for early detection and response to physiologic abnormalities in the ICU.

Abnormal vital signs are established predictors of clinical deterioration outcomes for hospitalized children outside of the ICU, forming the foundation of early warning scores.^24–29^ While single-center studies have identified prearrest hemodynamic patterns in pediatric ICUs, including hypotension and changes in heart rate variability, the prognostic value of derangements in children with cardiac arrest has not been evaluated.^4,7,30^ In our study, vital sign abnormalities – most often hypotension – preceded most cardiac arrests and independently predicted poor CPR outcomes. Moreover, specific combinations of vital signs such as tachycardia with tachypnea and narrowed pulse pressure, which align with common ICU pathophysiology (e.g., respiratory failure or shock), had particularly strong associations with poor outcomes. The link between specific patterns and outcomes could augment prearrest planning (e.g., epinephrine preparation for patients with evidence of shock) in high-risk situation awareness systems that have already shown success in reducing cardiac arrest events and improving outcomes.^31,32^

Similar to findings in adults with IHCA, we identified a dose–response relationship between the number of abnormal vital signs and outcomes.^33^ Although our multivariable regression models could not fully account for severity of illness, the persistent association between abnormal vitals and outcomes after adjusting for common prearrest illnesses and ICU therapies suggests these abnormalities carry prognostic information about response to CPR beyond the degree of critical illness. Cardiac arrest characteristics in children with abnormal vital signs also align with known factors associated with poor outcomes. They were more likely to be pulseless on CPR initiation,^34,35^ required longer duration of CPR,^36,37^ more often required ECPR,^38^ and had lower frequency of ROSC. Collectively, these data demonstrate that even among a cohort of ICU patients who all eventually required CPR, the degree to which their physiology was altered prearrest had clinical significance. Conversely, the absence of vital sign derangements in 37.7 % of patients, a group with improved post-arrest outcomes, also provides key prognostic context, potentially reflecting a greater amenability to successful resuscitation. These findings suggest that even intermittent prearrest vital sign data provide critical contextual information about the underlying physiologic state and its influence on resuscitation outcomes.

Current approaches to personalized CPR focus primarily on intra-arrest physiology, using hemodynamic targets to guide resuscitation efforts.^6,39^ The findings of this study point towards the potential value of integrating prearrest information into such resuscitation strategies. Importantly, this study examined single time point measurements and the majority of abnormal vital signs occurred in the hour closest to arrest. Future research should incorporate more high-fidelity vital sign data to evaluate whether longitudinal prearrest vital sign trends and responses to interventions could identify physiologic patterns predictive of poor response to conventional CPR. Such insights could inform decisions about ECPR or modifications of standardized resuscitation (e.g., alternative vasopressors for patients with prearrest hypotension despite high-dose catecholamines, prioritization of providing adequate oxygenation and ventilation to patients with signs of respiratory deterioration as the etiology of arrest). These approaches could improve outcomes by matching initial resuscitation efforts to patient-specific physiology rather than waiting to assess response to standard interventions.

This study has limitations. First, participation in the GWTG-R registry is voluntary and is generally comprised of hospitals with an interest in resuscitation quality. Hospitals included in this study were larger and had more arrests per year than those excluded. As such, our findings may not be generalizable to all care environments. Second, the optional nature of the vital sign section of the database introduces risk of bias due to variable vital sign reporting. Sites may report vital signs more frequently for patients who survive or obtain and report more vital signs for children with abnormal vital signs. We addressed this by restricting analysis to sites with consistent documentation and accounted for increasing vital sign sampling by limiting our primary analysis to only the single most recent abnormal vital signs. Residual confounding related to variation in abnormal vital sign timing is still possible. Third, a significant proportion of eligible patients had no vital signs reported. Systematic differences between patients included and excluded due to missing data primarily reflected variations in prearrest conditions and initial arrest characteristics. The direction of bias is unclear as vital sign reporting may be related to site resources or patient-specific factors such as severity of illness and outcomes of CPR. Reassuringly, sensitivity analyses incorporating broader inclusion criteria showed consistent associations.

## Conclusion

In this multicenter observational cohort study of pediatric IHCA, prearrest vital sign abnormalities were common and significantly associated with worse outcomes after adjusting for measures of severity of illness. An increasing number of abnormal vital signs were associated with progressively worse odds of survival in a dose–response fashion. These findings underscore the need for earlier physiologic detection and intervention in the ICU, supporting the development and refinement of ICU-based early warning approaches to improve outcomes.

## Supplementary Material

supplementary data

## Figures and Tables

**Table 1 –:** Characteristics of the study population.

	All Patients	Abnormal Vital Signs	No Abnormal Vital Signs	*p*
n (%)	2875	1790 (62.3)	1085 (37.7)	
Age (years), median [Q1, Q3]	1.0 [0.3, 7.0]	2.0 [0.6, 10.0]	0.4 [0.2, 2.0]	<0.01
Sex (female), n (%)	1344 (46.7)	845 (47.2)	499 (46.0)	0.60
Illness Category, n (%)				
Medical-Cardiac	542 (18.9)	337 (18.8)	205 (18.9)	<0.01
Medical-Non-cardiac	1417 (49.3)	930 (52.0)	487 (44.9)	
Surgical-Cardiac	713 (24.8)	400 (22.3)	313 (28.8)	
Surgical-Non-cardiac	203 (7.1)	123 (6.9)	80 (7.4)	
**Prearrest Conditions, n (%)**				
Respiratory Insufficiency	1791 (62.3)	1111 (62.1)	680 (62.7)	0.78
Hypotension/Hypoperfusion	823 (28.6)	632 (35.3)	191 (17.6)	<0.01
Metabolic/electrolyte abnormality	518 (18.0)	378 (21.1)	140 (12.9)	<0.01
Sepsis	441 (15.3)	341 (19.1)	100 (9.2)	<0.01
Pneumonia	236 (8.2)	153 (8.5)	83 (7.6)	0.44
Cyanotic Cardiac Malformation	705 (24.5)	403 (22.5)	302 (27.8)	<0.01
Acyanotic Cardiac Malformation	458 (15.9)	254 (14.2)	204 (18.8)	<0.01
Congenital Malformation (Non-Cardiac)	504 (17.5)	280 (15.6)	224 (20.6)	<0.01
Baseline depression in CNS function	466 (16.2)	291 (16.3)	175 (16.1)	0.97
Renal Insufficiency	342 (11.9)	249 (13.9)	93 (8.6)	<0.01
Metastatic/hematologic malignancy	197 (6.9)	164 (9.2)	33 (3.0)	<0.01
Acute CNS non-stroke event	189 (6.6)	130 (7.3)	59 (5.4)	0.07
**Prearrest Interventions, n (%)**				
Invasive assisted ventilation	2003 (69.7)	1263 (70.6)	740 (68.2)	0.20
Vasoactive agent	1499 (55.6)	1020 (60.6)	479 (47.2)	<0.01
**Arrest Characteristics, n (%)**				
Initial CPR Rhythm/Status				
Pulseless	1359 (47.3)	925 (51.7)	434 (40.0)	<0.01
Pulse with Poor Perfusion, Subsequently Pulseless	683 (23.8)	439 (24.5)	244 (22.5)	
Pulse with Poor Perfusion, Never Pulseless	833 (29.0)	426 (23.8)	407 (37.5)	
Duration of CPR, median [Q1,Q3]	12.0 [4.0, 34.0]	14.0 [5.0, 36.0]	10.0 [3.0, 29.0]	<0.01
**Arrest Outcomes, n (%)**				
ROSC	1896 (65.9)	1061 (59.3)	835 (77.0)	<0.01
ECPR	371 (12.9)	258 (14.4)	113 (10.4)	<0.01
Survival to hospital discharge	1356 (47.2)	689 (38.5)	667 (61.5)	<0.01

Descriptive characteristics of study population in primary analysis. Comparison of patients with no abnormal vitals and at least 1 abnormal vital (Heart rate or respiratory rate > 95 % for age, systolic blood pressure or diastolic blood pressure <5 % for age.) CNS = central nervous system. CPR = cardiopulmonary resuscitation. ROSC = sustained return of spontaneous circulation for >20 min. ECPR = extra-corporeal cardiopulmonary resuscitation.

**Table 2 –:** Frequency of abnormal and severely abnormal vital signs.

	Type of Vital Sign^a^	Number of Vital Signs Meeting Criteria			
		0	1	2	3
	Any	1085 (37.7)^b^	1069 (37.2)^b^	622 (21.6)^b^	99 (3.4)^b^
	HR (>95%)	na	325 (30.4)	526 (84.6)	99 (100)
Abnormal	RR (>95 %)	na	139 (13.0)	262 (42.1)	99 (100)
	SBP or DBP (<5%)	na	605 (56.6)	456 (73.3)	99 (100)
	Any	1727 (60.1)^c^	895 (31.1)^c^	233 (8.1)^c^	20 (0.7)^c^
	HR (> 99%)	na	281 (31.4)	205 (88.0)	20 (100)
Severely Abnormal	RR (>99 %)	na	95 (10.6)	109 (46.8)	20 (100)
	SBP or DBP (<1%)	na	519 (58.0)	152 (65.2)	20 (100)

Frequency of specific vital signs meeting abnormal or severely abnormal criteria categorized into how many concurrent vital signs were abnormal.

* Cell entries are the number of individuals in the column who had the specified abnormal or severely abnormal vital sign. Column percent.

HR = heart rate, RR = respiratory rate, SBP = systolic blood pressure, DBP = diastolic blood pressure.

a The type of vital sign with percentile cutoff for meeting abnormal or severely abnormal categories.

b Total number of individuals with abnormal vital signs falling into each category of number of abnormal vital signs, Row percent.

c Total number of individuals with severely abnormal vital signs falling into each category of number of severely abnormal vital signs, Row percent.

## Appendix A. Supplementary material

Supplementary data to this article can be found online at https://doi.org/10.1016/j.resuscitation.2025.110846.

## References

[1] Morgan RW, Kirschen MP, Kilbaugh TJ, Sutton RM, Topjian AA. Pediatric in-hospital cardiac arrest and cardiopulmonary resuscitation in the United States: a review. JAMA Pediatr 2021;175(3):293–302. 10.1001/jamapediatrics.2020.5039. 33226408 PMC8787313

[2] Gardner MM, Morgan RW, Reeder R, Trends in cardiac arrest outcomes & management in children with cardiac illness category compared to non-cardiac illness category: an analysis from the AHA get with the guidelines^®^-resuscitation registry. Resuscitation 2024. 10.1016/j.resuscitation.2024.110430. 40071352

[3] Holmberg MJ, Wiberg S, Ross CE, Trends in survival after pediatric in-hospital cardiac arrest in the United States. Circulation 2019;140(17):1398–408. 10.1161/CIRCULATIONAHA.119.041667. 31542952 PMC6803102

[4] Walker SB, Badke CM, Carroll MS, Novel approaches to capturing and using continuous cardiorespiratory physiological data in hospitalized children. Pediatr Res 2023;93(2):396–404. 10.1038/s41390-022-02359-3. 36329224

[5] Morgan RW, Berg RA, Reeder RW, The physiologic response to epinephrine and pediatric cardiopulmonary resuscitation outcomes. Crit Care 2023;27(1):105. 10.1186/s13054-023-04399-5. 36915182 PMC10012560

[6] Topjian AA, Raymond TT, Atkins D, Part 4: pediatric basic and advanced life support: 2020 American Heart Association guidelines for Cardiopulmonary Resuscitation and Emergency Cardiovascular Care. Circulation 2020;142(16_suppl_2):S469–523. 10.1161/CIR.0000000000000901. 33081526

[7] Erez E, Mazwi ML, Marquez AM, Moga MA, Eytan D. Hemodynamic patterns before inhospital cardiac arrest in critically ill children: an exploratory study. Crit Care Explor 2021;3(6):e0443. 10.1097/CCE.0000000000000443. 34151279 PMC8205221

[8] Mclean H, Wells L, Marler J. The effect of prearrest acid-base status on response to sodium bicarbonate and achievement of return of spontaneous circulation. Ann Pharmacother 2022;56(4):436–40. 10.1177/10600280211038393. 34353142

[9] Recommended Guidelines for Reviewing, Reporting, and Conducting Research on In-Hospital Resuscitation: The In-Hospital ‘Utstein Style’ | Circulation. https://www-ahajournals-org.proxy.library.upenn.edu/doi/full/10.1161/01.cir.95.8.2213. Accessed 13 November 2024.

[10] Nolan JP, Berg RA, Andersen LW, Cardiac Arrest and Cardiopulmonary Resuscitation Outcome reports: update of the Utstein Resuscitation Registry Template for In-Hospital Cardiac arrest: a Consensus Report from a Task Force of the International Liaison Committee on Resuscitation (American Heart Association, European Resuscitation Council, Australian and New Zealand Council on Resuscitation, Heart and Stroke Foundation of Canada, InterAmerican Heart Foundation, Resuscitation Council of Southern Africa, Resuscitation Council of Asia). Circulation 2019;140(18): e746–57. 10.1161/CIR.0000000000000710. 31522544

[11] Girotra S, Spertus JA, Li Y, Berg RA, Nadkarni VM, Chan PS. Survival trends in pediatric in-hospital cardiac arrests: an analysis from get with the guidelines-resuscitation. Circ Cardiovasc Qual Outcomes 2013;6(1):42–9. 10.1161/CIRCOUTCOMES.112.967968. 23250980 PMC3555689

[12] Jacobs I, Nadkarni V, Bahr J, Cardiac arrest and cardiopulmonary resuscitation outcome reports: update and simplification of the Utstein templates for resuscitation registries: a statement for healthcare professionals from a task force of the International Liaison Committee on Resuscitation (American Heart Association, European Resuscitation Council, Australian Resuscitation Council, New Zealand Resuscitation Council, Heart and Stroke Foundation of Canada, InterAmerican Heart Foundation, Resuscitation Councils of Southern Africa). Circulation 2004;110 (21):3385–97. 10.1161/01.CIR.0000147236.85306.15. 15557386

[13] Peberdy MA, Kaye W, Ornato JP, Cardiopulmonary resuscitation of adults in the hospital: a report of 14720 cardiac arrests from the National Registry of Cardiopulmonary Resuscitation. Resuscitation 2003;58(3):297–308. 10.1016/s0300-9572(03)00215-6. 12969608

[14] Sawyer T, McBride ME, Ades A, Considerations on the use of neonatal and pediatric resuscitation guidelines for hospitalized neonates and infants: on behalf of the American Heart Association Emergency Cardiovascular Care Committee and the American Academy of Pediatrics. Pediatrics 2023;153(1)e2023064681. 10.1542/peds.2023-064681. 38105696

[15] Bonafide CP, Brady PW, Keren R, Conway PH, Marsolo K, Daymont C. Development of heart and respiratory rate percentile curves for hospitalized children. Pediatrics 2013;131(4):e1150–7. 10.1542/peds.2012-2443. 23478871 PMC4074640

[16] Roberts JS, Yanay O, Barry D. Age-based percentiles of measured mean arterial pressure in pediatric patients in a hospital setting. Pediatr Crit Care Med 2020;21(9):e759–68. 10.1097/PCC.0000000000002495. 32740191

[17] Meaney PA, Nadkarni VM, Cook EF, Higher survival rates among younger patients after pediatric intensive care unit cardiac arrests. Pediatrics 2006;118(6):2424–33. 10.1542/peds.2006-1724. 17142528

[18] Federman M, Sutton RM, Reeder RW, Survival with favorable neurologic outcome and quality of cardiopulmonary resuscitation following in-hospital cardiac arrest in children with cardiac disease compared with noncardiac disease. Pediatr Crit Care Med 2024;25 (1):4–14. 10.1097/PCC.0000000000003368. 37678381 PMC10843749

[19] Reis AG, Nadkarni V, Perondi MB, Grisi S, Berg RA. A prospective investigation into the epidemiology of in-hospital pediatric cardiopulmonary resuscitation using the international Utstein reporting style. Pediatrics 2002;109(2):200–9. 10.1542/peds.109.2.200. 11826196

[20] López-Herce J, Del Castillo J, Matamoros M, Factors associated with mortality in pediatric in-hospital cardiac arrest: a prospective multicenter multinational observational study. Intensive Care Med 2013;39(2):309–18. 10.1007/s00134-012-2709-7. 23184036

[21] Knudson JD, Neish SR, Cabrera AG, Prevalence and outcomes of pediatric in-hospital cardiopulmonary resuscitation in the United States: an analysis of the Kids’ Inpatient Database*. Crit Care Med 2012;40(11):2940–4. 10.1097/CCM.0b013e31825feb3f. 22932398

[22] Van Rossum G, Drake FL. Python 3 Reference Manual. CreateSpace 2009.

[23] Seabold S, Perktold J. statsmodels: econometric and statistical modeling with python. 9th Python in science conference, 2010.

[24] Churpek MM, Adhikari R, Edelson DP. The value of vital sign trends for detecting clinical deterioration on the wards. Resuscitation 2016;102:1–5. 10.1016/j.resuscitation.2016.02.005. 26898412 PMC4834231

[25] Parshuram CS, Hutchison J, Middaugh K. Development and initial validation of the Bedside Paediatric Early Warning System score. Crit Care 2009;13(4):R135. 10.1186/cc7998. 19678924 PMC2750193

[26] Bonafide CP, Roland D, Brady PW. Rapid response systems 20 years later: new approaches, old challenges. JAMA Pediatrics 2016;170(8):729–30. 10.1001/jamapediatrics.2016.0398. 27322604 PMC4969134

[27] Agulnik A, Gossett J, Carrillo AK, Kang G, Morrison RR. Abnormal vital signs predict critical deterioration in hospitalized pediatric hematology-oncology and post-hematopoietic cell transplant patients. Front Oncol 2020;10:354. 10.3389/fonc.2020.00354. 32266139 PMC7105633

[28] Mayampurath A, Jani P, Dai Y, Gibbons R, Edelson D, Churpek MM. A vital sign-based model to predict clinical deterioration in hospitalized children. Pediatr Crit Care Med 2020;21(9):820–6. 10.1097/PCC.0000000000002414. 32511200 PMC7483876

[29] Jensen CS, Kirkegaard H, Aagaard H, Olesen HV. Clinical profile of children experiencing in-hospital clinical deterioration requiring transfer to a higher level of care. J Child Health Care 2019;23 (4):522–33. 10.1177/1367493518794400. 30124066

[30] Bose SN, Verigan A, Hanson J, Early identification of impending cardiac arrest in neonates and infants in the cardiovascular ICU: a statistical modelling approach using physiologic monitoring data. Cardiol Young 2019;29(11):1340–8. 10.1017/S1047951119002002. 31496467

[31] Dewan M, Soberano B, Sosa T, Assessment of a situation awareness quality improvement intervention to reduce cardiac arrests in the pediatric intensive care unit. Pediatr Crit Care Med 2022;23(1):4–12. 10.1097/PCC.0000000000002816. 34417417 PMC8738107

[32] Alten J, Cooper DS, Klugman D, Preventing cardiac arrest in the pediatric cardiac intensive care unit through multicenter collaboration. JAMA Pediatr 2022;176(10):1027–36. 10.1001/jamapediatrics.2022.2238. 35788631 PMC9257678

[33] Andersen LW, Kim WY, Chase M, The prevalence and significance of abnormal vital signs prior to in-hospital cardiac arrest. Resuscitation 2016;98:112–7. 10.1016/j.resuscitation.2015.08.016. 26362486 PMC4715919

[34] Khera R, Tang Y, Girotra S, Pulselessness after initiation of cardiopulmonary resuscitation for bradycardia in hospitalized children. Circulation 2019;140(5):370–8. 10.1161/CIRCULATIONAHA.118.039048. 31006260 PMC6663562

[35] Morgan RW, Reeder RW, Meert KL, Survival and hemodynamics during pediatric cardiopulmonary resuscitation for bradycardia and poor perfusion versus pulseless cardiac arrest. Crit Care Med 2020;48(6):881. 10.1097/CCM.0000000000004308. 32301844 PMC7895327

[36] O’Halloran A, Morgan RW, Kennedy K, Characteristics of pediatric in-hospital cardiac arrests and resuscitation duration. JAMA Netw Open 2024;7(7)e2424670. 10.1001/jamanetworkopen.2024.24670. 39078626 PMC11289702

[37] Matos RI, Watson RS, Nadkarni VM, Duration of cardiopulmonary resuscitation and illness category impact survival and neurologic outcomes for in-hospital pediatric cardiac arrests. Circulation 2013;127(4):442–51. 10.1161/CIRCULATIONAHA.112.125625. 23339874

[38] Loaec M, Himebauch AS, Reeder R, Outcomes of extracorporeal cardiopulmonary resuscitation for in-hospital cardiac arrest among Children with noncardiac illness categories. Crit Care Med 2024;52(4):551–62. 10.1097/CCM.0000000000006153. 38156912 PMC11810531

[39] The ICU-RESUS and Eunice Kennedy Shriver National Institute of Child Health, and Human Development Collaborative Pediatric Critical Care Research Network Investigator. Effect of physiologic point-of-care cardiopulmonary resuscitation training on survival with favorable neurologic outcome in cardiac arrest in pediatric ICUs: a randomized clinical trial. JAMA 2022;327(10):934–45. 10.1001/jama.2022.1738. 35258533 PMC8905390

