# Supplementary material for: Prearrest vital sign abnormalities are associated with adverse outcomes in pediatric ICU cardiac arrest: a get with the guidelines-resuscitation analysis
Source: Resuscitation. Author manuscript; Available in PMC 2025 Dec 1. (PMC12667964; doi:10.1016/j.resuscitation.2025.110846)
Supplement: supplementary data [file NIHMS2123491-supplement-supplementary_data.docx]

# Supplement

### Supplementary Table 1: The age-based percentile thresholds for defining abnormal (>95%) and severely abnormal (>99%) heart rate and respiratory rate

| Age Group (years) | HR | | RR | |
| --- | --- | --- | --- | --- |
|  | **95%** | **99%** | **95%** | **99%** |
| 0-0.25 | 171 | 186 | 62 | 76 |
| 0.25-0.50 | 167 | 182 | 58 | 71 |
| 0.50-0.75 | 163 | 178 | 54 | 67 |
| 0.75-1 | 160 | 176 | 51 | 63 |
| 1.00-1.50 | 157 | 173 | 48 | 60 |
| 1.50-2.00 | 154 | 170 | 45 | 57 |
| 2.00-3.00 | 150 | 167 | 42 | 54 |
| 3.00-4.00 | 146 | 164 | 40 | 52 |
| 4.00-6.00 | 142 | 161 | 37 | 50 |
| 6.00-8.00 | 137 | 155 | 35 | 46 |
| 8.00-12.00 | 129 | 147 | 31 | 41 |
| 12.00-15.00 | 121 | 138 | 28 | 35 |
| 15.00-18.00 | 115 | 132 | 26 | 32 |

Age-based percentile thresholds for Heart Rate (HR) and Respiratory Rate (RR) from Bonafide CP, Brady PW, Keren R, Conway PH, Marsolo K, Daymont C. Development of Heart and Respiratory Rate Percentile Curves for Hospitalized Children. *Pediatrics*. 2013;131(4):e1150-e1157. doi:[10.1542/peds.2012-2443](https://doi.org/10.1542/peds.2012-2443)

### Supplementary Table 2: The age-based percentile thresholds for defining abnormal (<5%) and severely abnormal (<1%) systolic blood pressure and diastolic blood pressure

| Age Group (years) | SBP | | DBP | |
| --- | --- | --- | --- | --- |
|  | **1%** | **5%** | **1%** | **5%** |
| 0.00–0.08 | 50 | 58 | 23 | 29 |
| 0.08-0.25 | 53 | 62 | 24 | 31 |
| 0.25-0.50 | 59 | 68 | 26 | 33 |
| 0.50-1.00 | 68 | 77 | 29 | 36 |
| 1.00-2.00 | 77 | 86 | 32 | 40 |
| 2.00-3.00 | 77 | 86 | 33 | 40 |
| 3.00-4.00 | 75 | 84 | 32 | 39 |
| 4.00-5.00 | 75 | 84 | 32 | 40 |
| 5.00-6.00 | 76 | 85 | 33 | 41 |
| 6.00-7.00 | 79 | 87 | 34 | 42 |
| 7.00-8.00 | 81 | 89 | 35 | 42 |
| 8.00-9.00 | 82 | 90 | 35 | 43 |
| 9.00-10.00 | 83 | 91 | 35 | 43 |
| 10.00-11.00 | 83 | 91 | 35 | 42 |
| 11.00-12.00 | 83 | 91 | 35 | 42 |
| 12.00-13.00 | 83 | 91 | 35 | 42 |
| 13.00-14.00 | 83 | 91 | 35 | 42 |
| 14.00-15.00 | 83 | 92 | 35 | 42 |
| 15.00-16.00 | 83 | 92 | 35 | 43 |
| 16.00-17.00 | 83 | 93 | 35 | 43 |
| 17.00-18.00 | 84 | 94 | 35 | 44 |

Age-based percentile thresholds for systolic blood pressure (SBP) and diastolic blood pressure (DBP) from Roberts JS, Yanay O, Barry D. Age-Based Percentiles of Measured Mean Arterial Pressure in Pediatric Patients in a Hospital Setting. *Pediatr Crit Care Med*. 2020;21(9):e759-e768. doi:[10.1097/PCC.0000000000002495](https://doi.org/10.1097/PCC.0000000000002495)

## Supplementary Table 3: Characteristics of Patients Meeting Inclusion Criteria: Comparison Between Included and Excluded Cases Due to Missing Vital Signs

|  | All Patients Meeting Inclusion Criteria | Included | Excluded | *SMD* | *P* |
| --- | --- | --- | --- | --- | --- |
| n (%) | 6470 | 2875 (44.4) | 3595 (55.6) |  |  |
| Age (years), median [Q1, Q3] | 1.0 [0.3,7.0] | 1.0 [0.3,7.0] | 1.0 [0.3,7.0] | 0.004 | 0.819 |
| Sex (female), n (%) | 2996 (46.3) | 1344 (46.7) | 1652 (46.0) | 0.019 | 0.761 |
| Illness Category, n (%) |  | | | | |
| Medical-Cardiac | 1195 (18.5) | 542 (18.9) | 653 (18.2) | 0.018 | <0.01 |
| Medical-Non-cardiac | 3299 (51.0) | 1417 (49.3) | 1882 (52.4) | 0.061 |  |
| Surgical-Cardiac | 1474 (22.8) | 713 (24.8) | 761 (21.2) | 0.087 |  |
| Surgical-Non-cardiac | 456 (7.0) | 203 (7.1) | 253 (7.0) | 0.001 |  |
| **Prearrest Conditions, n (%)** |  | | | | |
| Respiratory Insufficiency | 4144 (64.0) | 1791 (62.3) | 2353 (65.5) | 0.066 | 0.009 |
| Hypotension/Hypoperfusion | 1788 (27.6) | 823 (28.6) | 965 (26.8) | 0.040 | 0.117 |
| Metabolic/electrolyte abnormality | 1219 (18.8) | 518 (18.0) | 701 (19.5) | 0.038 | 0.138 |
| Sepsis | 984 (15.2) | 441 (15.3) | 543 (15.1) | 0.007 | 0.821 |
| Pneumonia | 607 (9.4) | 236 (8.2) | 371 (10.3) | 0.073 | 0.004 |
| Cyanotic Cardiac Malformation | 1415 (21.9) | 705 (24.5) | 710 (19.7) | 0.115 | <0.001 |
| Acyanotic Cardiac Malformation | 1055 (16.3) | 458 (15.9) | 597 (16.6) | 0.018 | 0.486 |
| Congenital Malformation (Non-Cardiac) | 1133 (17.5) | 504 (17.5) | 629 (17.5) | 0.001 | 0.998 |
| Baseline depression in CNS function | 939 (14.5) | 466 (16.2) | 473 (13.2) | 0.086 | 0.001 |
| Renal Insufficiency | 713 (11.0) | 342 (11.9) | 371 (10.3) | 0.050 | 0.049 |
| Metastatic/hematologic malignancy | 389 (6.0) | 197 (6.9) | 192 (5.3) | 0.063 | 0.013 |
| Acute CNS non-stroke event | 428 (6.6) | 189 (6.6) | 239 (6.6) | 0.003 | 0.945 |
| **Prearrest Interventions, n (%)** |  | | | |  |
| Invasive assisted ventilation | 4518 (69.8) | 2003 (69.7) | 2515 (70.0) | 0.006 | 0.823 |
| Vasoactive agent | 3274 (50.6) | 1561 (54.3) | 1713 (47.6) | 0.133 | <0.001 |
| **Arrest Characteristics n (%)** |  | | | | |
| Initial Condition |  | | | | |
| Pulseless | 3084 (47.7) | 1359 (47.3) | 1725 (48.0) | 0.014 | <0.01 |
| Pulse with Poor Perfusion,  Subsequently Pulseless | 1514 (23.4) | 683 (23.8) | 831 (23.1) | 0.015 |  |
| Pulse with Poor Perfusion, Never  Pulseless | 1853 (28.6) | 833 (29.0) | 1020 (28.4) | 0.013 |  |
| Duration of CPR, median [Q1,Q3] | 12.0 [4.0,33.0] | 12.0 [4.0,34.0] | 12.0 [4.0,32.0] | 0.028 | 0.537 |
| **Arrest Outcomes, n (%)** |  | | | | |
| ROSC | 4922 (76.1) | 2218 (77.1) | 2704 (75.2) | 0.045 | 0.075 |
| ECPR | 108 (1.7) | 49 (1.7) | 59 (1.6) | 0.005 | 0.921 |
| Survival | 2956 (45.7) | 1356 (47.2) | 1600 (44.5) | 0.053 | 0.035 |

Descriptive characteristics of all patients that met inclusion criteria. Comparison of patients who were included in final analysis and those that were excluded due to missing vital signs. ECPR = extra-corporeal cardiopulmonary resuscitation. ROSC = sustained return of spontaneous circulation for >20 minutes. Survival = survival to hospital discharge. CPR = cardiopulmonary resuscitation. SMD = standardized mean difference.

## Supplementary Table 4: Comparison of sites that were included and excluded based on vital sign reporting frequency

|  | All Sites | Included Sites | Excluded Sites | *p* |
| --- | --- | --- | --- | --- |
|  | 145 | 86 (59.3%) | 59 (40.7%) |  |
| Pediatric Only Hospital n (%) | 98 (67.6%) | 56 (65.1%) | 42 (71.2%) | 0.674 |
| Number of Pediatric Beds, n (%) |  | | | |
| <100 | 107 (73.8%) | 58 (67.4%) | 49 (83.1%) | 0.029 |
| >=100 | 33 (22.8%) | 26 (30.2%) | 7 (11.9%) |  |
| Unknown | 5 (3.4%) | 2 (2.3%) | 3 (5.1%) |  |
| Teaching Status, n (%) |  | | | |
| Major Teaching Hospital | 56 (38.6%) | 31 (36.0%) | 25 (42.4%) | 0.854 |
| Minor Teaching Hospital | 59 (40.7%) | 36 (41.9%) | 23 (39.0%) |  |
| Non-Teaching Hospital | 7 (4.8%) | 4 (4.7%) | 3 (5.1%) |  |
| Unknown | 23 (15.9%) | 15 (17.4%) | 8 (13.6%) |  |
| Total Arrests*, median [IQR] | 9.0 (3.00 - 41.00) | 19.5 (5.25 - 65.0) | 5.0 (2.0 - 13.5) | <0.01 |
| Percent of Total Arrests* with Vitals, median [IQR] | 77% (20%-100%) | 95% (79%-100%) | 5% (0%-33%) | <0.01 |
| Average yearly arrests per site, median [IQR] | 2.33 (1.43 - 6.60) | 4.46 (1.74 - 10.45) | 1.67 (1.00 - 3.00) | <0.01 |
| Average yearly arrests with vital signs per site, median [IQR] | 1.33 (0.33 - 4.75) | 3.76 (1.58 - 8.64) | 0.25 (0.00 - 0.68) | <0.01 |

Site characteristics, including cardiac arrest reporting frequency, stratified by sites that met and did not meet inclusion criteria (specifically sites that report at least 1 set of vital signs (HR+RR+SBP+DBP) for greater than 50% of eligible in-hospital cardiac arrest cases per year.

*All counts of arrest events represent counts of cardiac arrests that meet all other study inclusion criteria.

## Supplementary Table 5: Association of Covariates with Survival to Hospital Discharge and Return of Spontaneous Circulation in the Primary Multivariable Model

| Variable | Survival  aOR (95% CI) | ROSC  aOR (95% CI) |
| --- | --- | --- |
| Age (years) | 0.95 (0.93 - 0.97) | 0.96 (0.95 - 0.98) |
| Illness Category |  |  |
| Medical-Cardiac | 1.09 (0.86 - 1.40) | 1.26 (0.99 - 1.59) |
| Medical-Noncardiac | Ref | 1.26 (0.96 - 1.66) |
| Surgical-Cardiac | 2.08 (1.61 - 2.68) | Ref |
| Surgical-Noncardiac | 1.32 (0.94 - 1.87) | 1.66 (1.23 - 2.24) |
| Respiratory Insufficiency | 1.07 (0.87 - 1.31) | 1.32 (1.11 - 1.58) |
| Hypotension/Hypoperfusion | 0.71 (0.58 - 0.88) | 0.65 (0.53 - 0.81) |
| Metabolic/electrolyte abnormality | 0.57 (0.43 - 0.74) | 0.70 (0.55 - 0.89) |
| Sepsis | 0.60 (0.46 - 0.77) | 0.82 (0.66 - 1.03) |
| Pneumonia | 1.82 (1.29 - 2.58) | 1.25 (0.89 - 1.76) |
| Cyanotic Cardiac Malformation | 0.69 (0.51 - 0.94) | 0.70 (0.54 - 0.91) |
| Acyanotic Cardiac Malformation | 1.46 (1.07 - 1.98) | 1.07 (0.85 - 1.35) |
| Congenital Malformation (Non-Cardiac) | 0.99 (0.81 - 1.21) | 1.04 (0.84 - 1.28) |
| Baseline depression in CNS function | 1.07 (0.84 - 1.37) | 1.25 (1.01 - 1.56) |
| Renal Insufficiency | 0.44 (0.33 - 0.58) | 0.88 (0.70 - 1.10) |
| Vasoactive agent | 0.64 (0.52 - 0.78) | 0.63 (0.53 - 0.76) |
| Invasive assisted ventilation | 0.70 (0.56 - 0.87) | 0.96 (0.79 - 1.16) |

The table displays the adjusted odds ratios (aOR) and 95% confidence intervals (CI) for all covariates from the primary multivariable GEE model, using presence of any abnormal vital sign as the representative exposure. Survival = Survival to Hospital Discharge. ROSC = Return of Spontaneous Circulation.

## Supplementary Figure 1: The Cardiac Arrest Prearrest Case Report Form Section Including Prearrest Vital signs
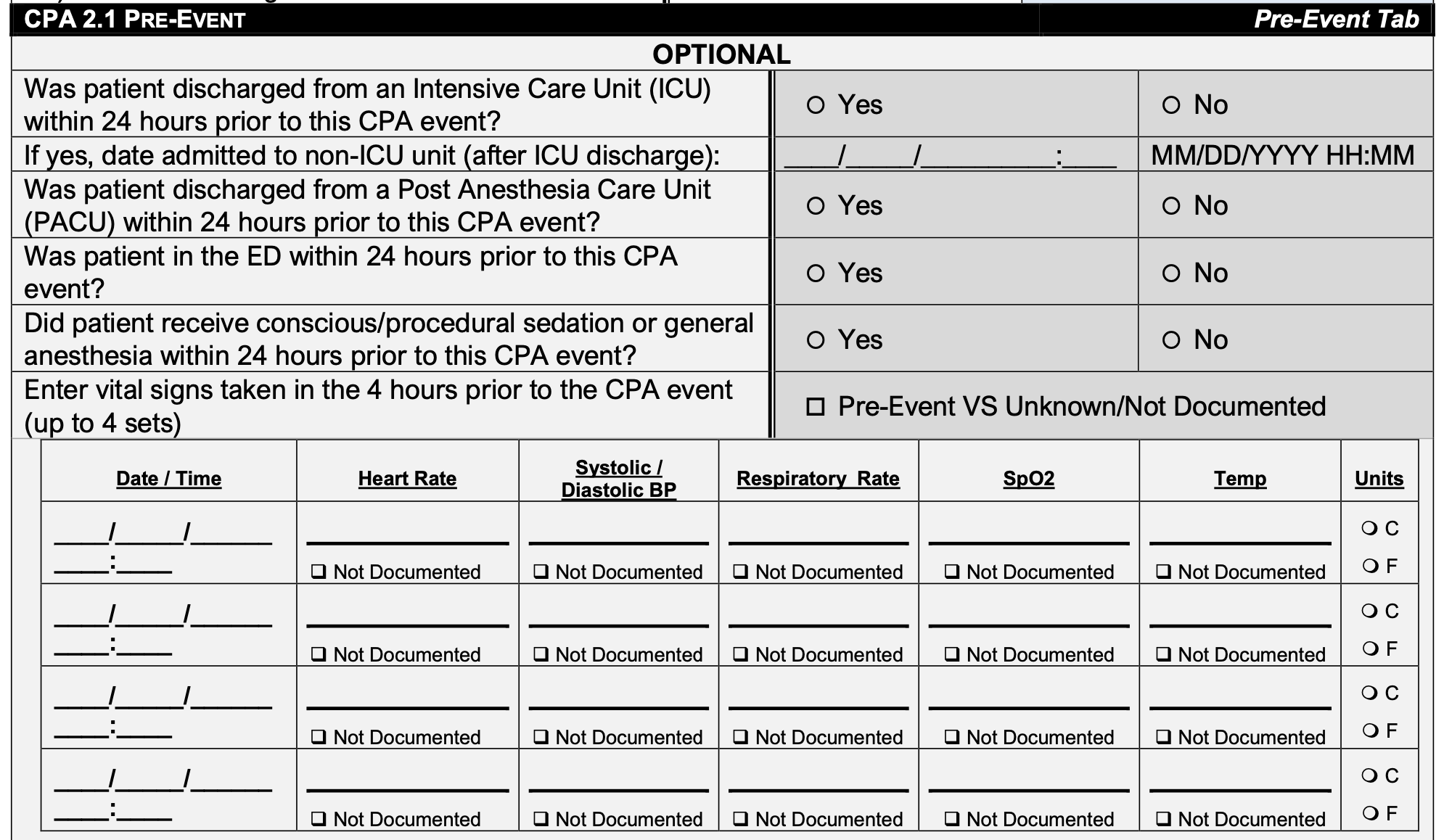


## Supplementary Figure 2: Temporal Distribution of Most Recent Vital Sign Prior to Cardiac Arrest and Proportion that are Abnormal

##
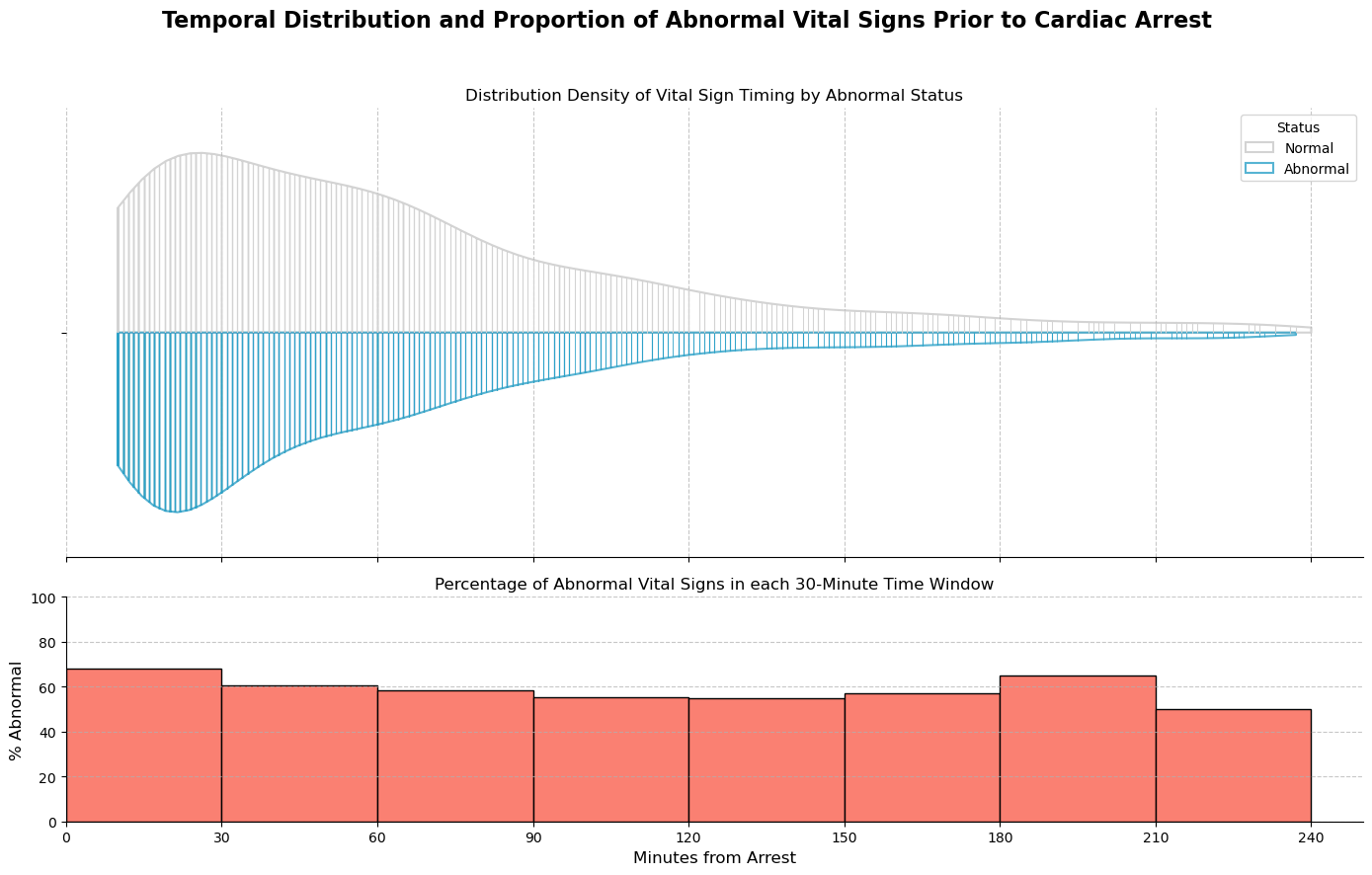


The top panel displays the distribution density of the timing of the most recent vital prior to cardiac arrest for each arrest that had a valid set of vital signs in the 10 minutes to 240 minutes prior to arrest. Each vertical line within the plot represents a reported vital sign time. The lines represent each individual vital sign sample. The bottom panel displays the percent abnormal of vital signs available in that time interval (E.g. 0 to 30 minutes).

##
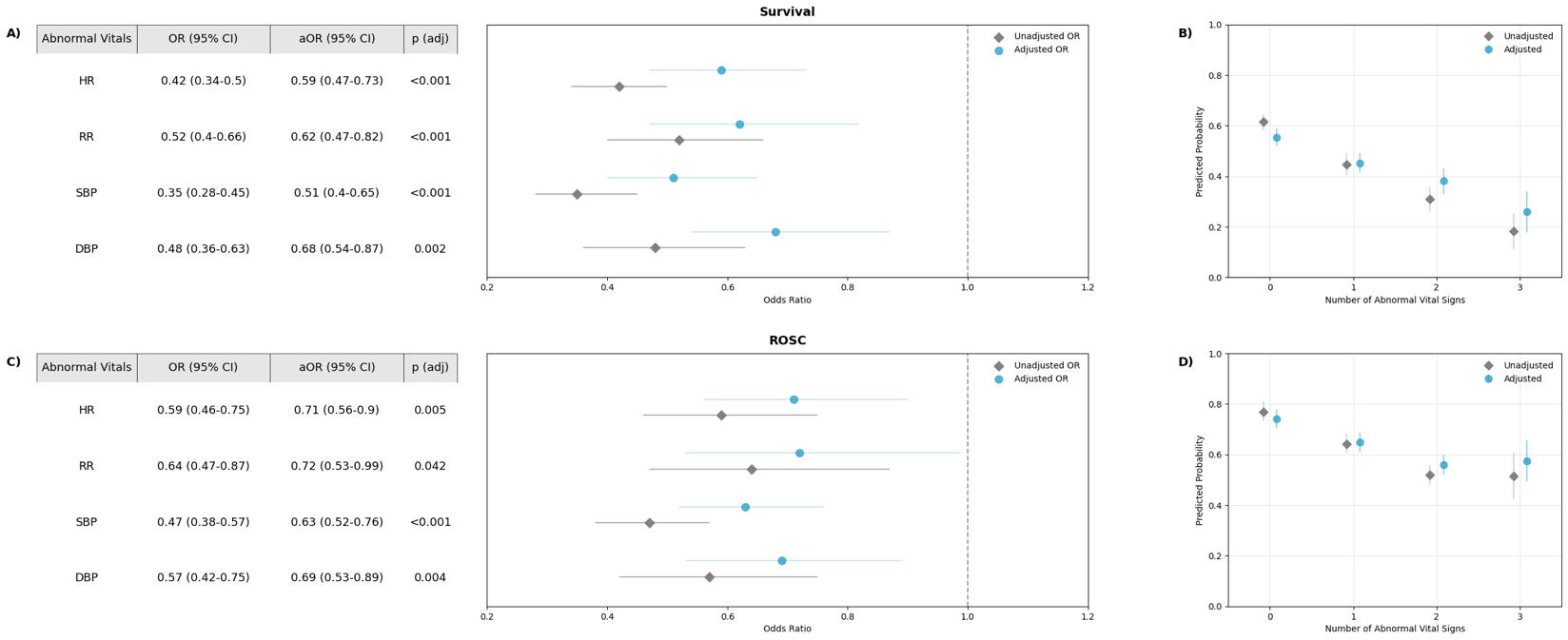
Supplementary Figure 3: Association of the presence of severe abnormal vital signs with outcomes.

Forrest plots (A and C) show the association between severely abnormal vital sign exposures for survival (A) and ROSC (C). Adjusted odds ratios account for age, illness category, prearrest conditions, and ICU intervention. Error bars represent the 95% confidence interval. P-values report the results of the adjusted odds ratios.

Panels B and D show the association between the number of severely abnormal vital signs and predicted probability of survival (B) and ROSC (D) after adjustment for age, illness category, prearrest conditions, and ICU intervention. Error bars represent the 95% confidence interval.

HR = Heart rate (abnormal defined as >99% for age). RR = Respiratory rate (abnormal defined as >99% for age). SBP = Systolic blood pressure (abnormal defined as <1% for age), DBP = Diastolic blood pressure (abnormal defined as <1% for age).

## Supplementary Figure 4: Association of abnormal vital signs with outcomes amongst the earliest vital signs recorded.


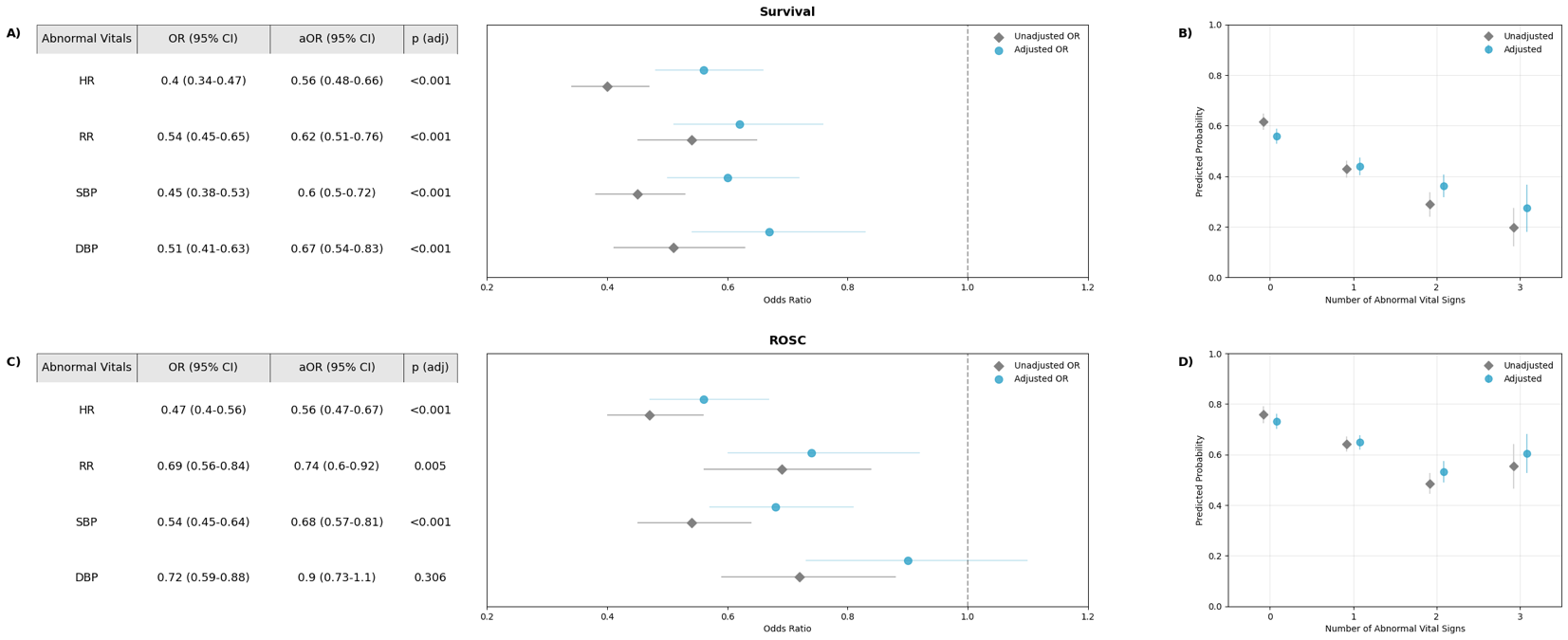


In contrast to the primary analysis: the set of vital sign farthest from arrest in the 10 minutes to 240 minutes prior to arrest were evaluated for meeting abnormal criteria.

Forrest plots (A and C) show the association between earliest abnormal vital sign exposures for survival (A) and ROSC (C). Adjusted odds ratios account for age, illness category, prearrest conditions, and ICU intervention. Error bars represent the 95% confidence interval. P-values report the results of the adjusted odds ratios.

Panels B and D show the association between the number of abnormal vital signs and predicted probability of survival (B) and ROSC (D) after adjustment for age, illness category, prearrest conditions, and ICU intervention. Error bars represent the 95% confidence interval.

HR = Heart rate (abnormal defined as >95% for age). RR = Respiratory rate (abnormal defined as >95% for age). SBP = Systolic blood pressure (abnormal defined as <5% for age), DBP = Diastolic blood pressure (abnormal defined as <5% for age). Pulse pressure = Difference between SBP and DBP (abnormal defined as <20 mmHg).

##
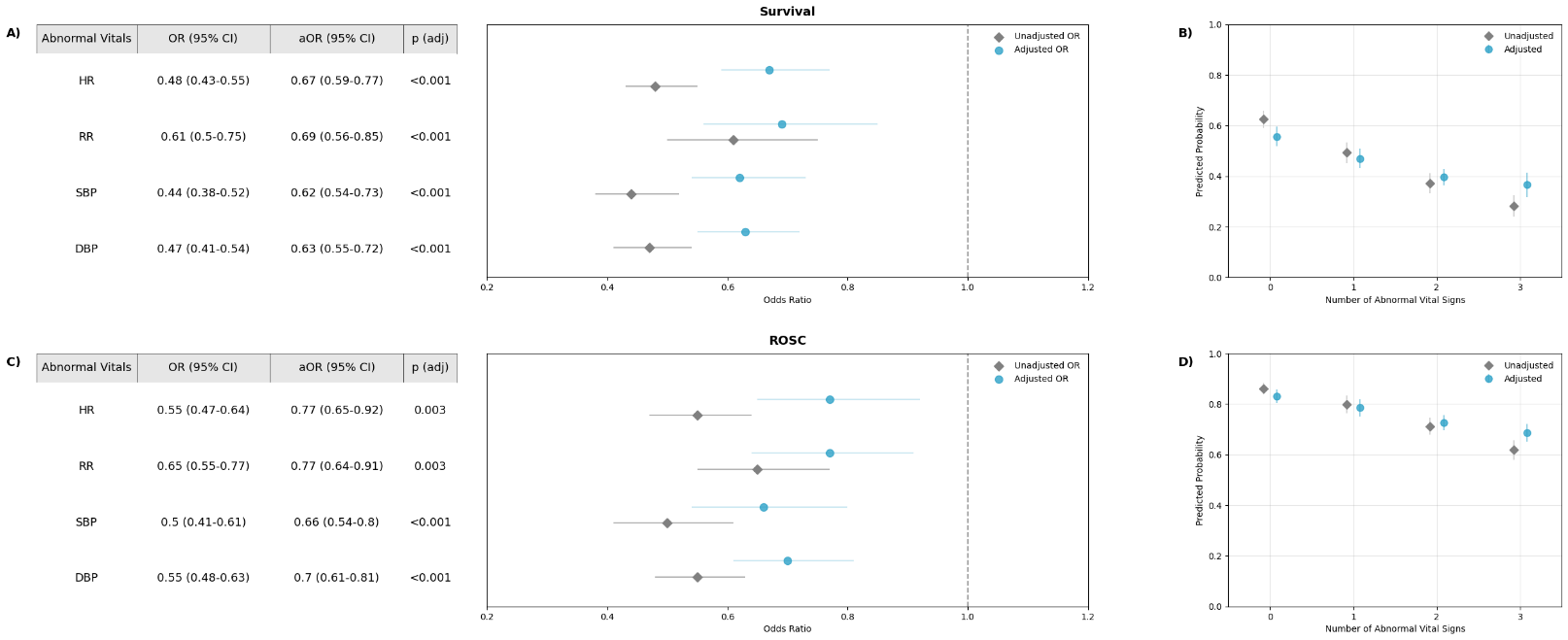
Supplementary Figure 5: Association of the presence of abnormal vital signs at any time with outcomes.

In contrast to the primary analysis: all vital signs reported for a patient in the 0 to 4 hours prior to arrest were evaluated for presence of abnormality.

Forrest plots (A and C) show the association between earliest abnormal vital sign exposures for survival (A) and ROSC (C). Adjusted odds ratios account for age, illness category, prearrest conditions, and ICU intervention. Error bars represent the 95% confidence interval. P-values report the results of the adjusted odds ratios.

Panels B and D show the association between the number of abnormal vital signs and predicted probability of survival (B) and ROSC (D) after adjustment for age, illness category, prearrest conditions, and ICU intervention. Error bars represent the 95% confidence interval.

HR = Heart rate (abnormal defined as >95% for age). RR = Respiratory rate (abnormal defined as >95% for age). SBP = Systolic blood pressure (abnormal defined as <5% for age), DBP = Diastolic blood pressure (abnormal defined as <5% for age). Pulse pressure = Difference between SBP and DBP (abnormal defined as <20 mmHg).
